# Circulating branched-chain amino acids and the risk of major adverse cardiovascular events in the UK biobank

**DOI:** 10.3389/fendo.2025.1510910

**Published:** 2025-02-20

**Authors:** Wanwan Sun, Ruilang Lin, Yiming Li, Ye Yao, Bin Lu, Yongfu Yu

**Affiliations:** ^1^ Department of Endocrinology, Huashan Hospital Affiliated to Fudan University, Shanghai, China; ^2^ Department of Biostatistics, School of Public Health, The Key Laboratory of Public Health Safety of Ministry of Education, Fudan University, Shanghai, China; ^3^ Department of Endocrinology, Huadong Hospital Affiliated to Fudan University, Shanghai, China

**Keywords:** branched-chain amino acids (BCAAs), major adverse cardiovascular events (MACE), UK Biobank, isoleucine, leucine, valine

## Abstract

**Objective:**

To investigate the relationship between circulating branched-chain amino acids (BCAAs) and the risk of major adverse cardiovascular events (MACE) in a national population-based cohort study.

**Methods:**

UK Biobank, a prospective study involving 22 recruitment centers across the United Kingdom. For this analysis, we included 266,840 participants from the UK Biobank who had available BCAA data and no history of MACE at baseline. Cox regression analysis was conducted to evaluate these associations, adjusting for potential confounders.

**Results:**

During a 13.80 ± 0.83-year follow-up, 52,598 participants experienced MACE, with the incidence of MACE increasing progressively across quintiles of circulating BCAAs, isoleucine, leucine, and valine. Overall, the fifth quintile exhibited a 7-12% higher MACE risk compared to the second quintile. In males, BCAAs were not associated with MACE risk. However, increased risks were observed for isoleucine (8-12% in higher quintiles), leucine (9% in the first quintile and 6% in the fifth quintile), and valine (8% in the first quintile). In females, higher quintiles of BCAAs, isoleucine, leucine, and valine were associated with increased MACE risk, ranging from 9% to 12%. Among participants under 65y, higher quintiles of BCAAs, isoleucine, and leucine were associated with increased MACE risk, while valine showed no significant association. No association was found in participants aged 65 and older. These analyses were adjusted for multiple potential confounders.

**Conclusion:**

Generally, higher levels of BCAAs, isoleucine, leucine, and valine were associated with an increased risk of MACE, except in participants older than 65. Additionally, in males, the lowest quintiles of leucine and valine were also associated with an increased risk of MACE.

## 1 Introduction

Branched-chain Amino Acids (BCAAs) — namely isoleucine, leucine, and valine — are essential amino acids that play a crucial role in metabolic homeostasis through nutritional signaling (1). Elevated levels of BCAAs and their related metabolites are now recognized as metabolic markers for obesity, insulin resistance, and type 2 diabetes in humans (2, 3). Given the close link between metabolic disorders and the pathogenesis of cardiovascular disease (CVD), research suggests that BCAAs may directly contribute to heart failure (HF) (4), vascular disease (5, 6), hypertension (7), and arrhythmias (8).

Currently, the mechanisms underlying the association between BCAAs and cardiovascular disease (CVD) are not fully understood. Known mechanisms include the activation of the serine/threonine protein kinase mTOR by BCAAs (9), mitochondrial dysfunction (10), alterations in cardiac substrate utilization, and platelet activation (11).

Additional studies have found that cardiovascular disease (CVD) can induce changes in tissues that regulate BCAA homeostasis, such as skeletal muscle, liver, and adipose tissue. These changes may contribute to elevated circulating level of BCAAs in individuals with CVD (12). Therefore, further research is needed to elucidate the causal relationship between BCAA dysregulation and CVD.

Currently, there are limited large-scale, prospective clinical studies investigating the causal relationship between BCAAs - including isoleucine, leucine, and valine - and cardiovascular disease (CVD) risk (13). While most studies suggest that high levels of amino acids (AAs) predict increased CVD risk (14), many of these studies have primarily focused on aromatic amino acids and specific populations (such as heart failure patients, women, and the elderly) (13, 15–18), and typically involve relatively small sample sizes. Some studies have even suggested that reduced BCAAs levels are associated with an increased risk of major adverse cardiovascular events (MACE), particularly in populations of elderly men over 70 years of age (17). Given the notable differences in BCAA levels between men and women, as well as across age groups, there is a need for large-sample studies that systematically explore how BCAA levels correlate with CVD risk across various demographics.

Our study aimed to investigate the relationship between baseline BCAAs and the risk of major adverse cardiovascular events (MACE) in a large prospective cohort. Additionally, we explored whether gender and age influence this relationship.

## 2 Materials and methods

### 2.1 Study design and sample

UK Biobank recruited participants aged between 40 and 70 years from across the UK between 2006 to 2010 (19). At enrollment, comprehensive data were collected on sociodemographic factors (e.g., age, sex), lifestyle behaviors (smoking and drinking status, diet, and physical activity level), medical history, and genetic information through touchscreen questionnaires, physical examinations, and sample analyses. Baseline biochemical assays included amino acids, HbA1c, and blood lipids measurements.

Health-related outcomes were monitored through regular linkages with various national datasets, including primary care records, hospital admissions, and mortality registries (20).

### 2.2 Standard protocol approvals, registrations, and patient consents

Assessments were conducted at 22 centers across 22 Scotland, England, and Wales as part of the UK Biobank study. The assessment process consisted of five components: written consent, touchscreen questionnaires (including detailed dietary recall), face-to-face interviews with study nurses, physical measurements (such as hand grip strength, spirometry, and bone density scans), and collection of blood, urine, and saliva samples.

The UK Biobank database contains information from 502,359 participants. In this study, participants who had prevalent MACE at the time of recruitment (n=13,546) or had missing values for BCAAs, isoleucine, leucine, and valine (n=222,003) were excluded from the analysis. Ultimately, 266,840 participants were included in the study. The UK Biobank has obtained ethical approval from the NHS National Research Ethics Service (16/NW/0274), and all participants provided informed consent before data collection.

### 2.3 Outcome measure: MACE

The primary outcome of this study was the incidence of MACE, defined as a composite endpoint including cardiovascular death, nonfatal myocardial infarction, nonfatal stroke, coronary revascularization, and hospitalization for unstable angina. Cases were identified using hospital inpatient records (primary or secondary hospital diagnosis) or death registry records (underlying or contributory cause of death). Diagnosis were based on the International Cleucinesification of Diseases coding system (ICD-9 and ICD-10).

### 2.4 BCAAs and covariates

Given the inconsistencies in prior research regarding the correlation between BCAAs levels and the risk of MACE, particularly in males, we hypothesize a potential J-shaped relationship. To more effectively explore this relationship, we have categorized the levels of BCAAs, isoleucine, leucine, and valine into quintiles,Q2 was used as the reference category. Covariates included gender, age, body mass index (BMI) categorized as underweight (<18.5kg/m^2^), normal (18.5-23.9 kg/m^2^), overweight (24.0-28.0 kg/m^2^), and obese (≥28.0 kg/m^2^) (21). Other covariates included HbA1c, Low-density lipoprotein cholesterol(LDL-c), systolic blood pressure(SBP), smoking and drinking status (never, current, and past), and physical activity level (measured in MET).

### 2.5 Statistical analysis

Cumulative incidence of MACE was computed according to maternal HDP status. We examined the association of BCAAs level - including isoleucine, leucine, and valine - with MACE using a Cox regression model with follow-up time as the time scale. Adjusted hazard ratios and corresponding 95% confidence intervals were calculated after adjusting for gender, age, body mass index (BMI)(underweight: <18.5, normal: 18.5-23.9, overweight: 24.0-28.0, obese: ≥28.0), HbA1c, LDL-c, SBP, smoking and drinking status(never, current and past), and activity (Metabolic Equivalent of Task, MET).

To evaluate the influence of gender and age on the relationship between BCAAs and MACE, we conducted separate analyses for men and women, as well as for individuals aged 65 and above compared to those below this age threshold.

## 3 Results

### 3.1 Baseline characteristics

A total of 266840 individuals were included in the final study, 121,067(45.37%) males and 145,773 (54.63%) females. The baseline average age was 56.95 ± 8.08 yrs. During a follow-up period of 13.80 ± 0.83 years, MACE occurred in 52,598(10.47%) participants.

Baseline characteristics showed that BMI, HbA1c, triglyceride, and C-reactive protein (CRP) increased progressively across the quintile of BCAAs, while HDL-C level decreased across the quintiles. Age, SBP, and DBP (diastolic blood pressure) showed consistent trends(**Table 1**). Similar trends were observed for BMI, blood pressure, HbA1c, triglycerides, CRP, HDL, Age, SBP, and DBP across the quintiles of isoleucine, leucine, and valine.

**Table 1:** Baseline characteristics according to quintiles of BCAAs, isoleucine, leucine and valine.

	Quintiles of BCAAs					
Characteristics	Q1	Q2	Q3	Q4	Q5	P value
Age (years)	56.38 ± 8.30	57.12 ± 8.09	57.17 ± 8.04	57.10 ± 8.00	57.00 ± 7.94	<.0001
BMI (kg/m^2^)	25.54 ± 4.33	26.76 ± 4.53	27.58 ± 4.65	28.28 ± 4.71	28.93 ± 4.86	<.0001
SBP (mmHg)	137.34 ± 20.47	139.47 ± 19.97	140.45 ± 19.58	140.98 ± 19.15	140.68 ± 18.71	<.0001
DBP (mmHg)	80.54 ± 10.62	81.96 ± 10.68	82.71 ± 10.61	83.19 ± 10.63	82.65 ± 10.59	<.0001
HbA1c (%)	34.60 (32.20-37.00)	35.00 (32.60-37.40)	35.20 (32.80-37.80)	35.40 (33.00-38.20)	35.80 (33.20-38.90)	<.0001
Total cholesterol (mmol/L)	5.71 ± 1.10	5.75 ± 1.12	5.74 ± 1.13	5.71 ± 1.15	5.66 ± 1.16	<.0001
Triglyceride (mmol/L)	1.11 (0.84-1.52)	1.31 (0.97-1.81)	1.48 (1.08-2.07)	1.70 (1.22-2.38)	2.03 (1.43-2.89)	<.0001
HDL-C (mmol/L)	1.63 ± 0.40	1.52 ± 0.38	1.43 ± 0.36	1.36 ± 0.34	1.30 ± 0.33	<.0001
LDL-C (mmol/L)	3.50 ± 0.84	3.59 ± 0.86	3.62 ± 0.86	3.61 ± 0.87	3.56 ± 0.87	<.0001
CRP (mmol/L)	1.06 (0.52-2.30)	1.24 (0.61-2.63)	1.37 (0.68-2.80)	1.46 (0.74-2.97)	1.54 (0.79-3.05)	<.0001
	Quintiles of Isoleucine					
	Q1	Q2	Q3	Q4	Q5	
Age (years)	56.57 ± 8.27	57.10 ± 8.09	57.12 ± 8.01	56.94 ± 8.04	57.04 ± 7.99	<.0001
BMI (kg/m^2^)	25.90 ± 4.43	26.87 ± 4.61	27.55 ± 4.69	28.16 ± 4.79	28.59 ± 4.83	<.0001
SBP (mmHg)	137.95 ± 20.30	139.62 ± 20.01	140.47 ± 19.69	140.69 ± 19.18	140.19 ± 18.81	<.0001
DBP (mmHg)	81.00 ± 10.58	82.08 ± 10.69	82.77 ± 10.68	82.99 ± 10.65	82.21 ± 10.61	<.0001
HbA1c (%)	34.70 (32.30-37.10)	35.00 (32.60-37.50)	35.20 (32.80-37.80)	35.40 (32.90-38.20)	35.70 (33.20-38.70)	<.0001
Total cholesterol (mmol/L)	5.76 ± 1.11	5.76 ± 1.12	5.73 ± 1.13	5.68 ± 1.14	5.64 ± 1.16	<.0001
Triglyceride (mmol/L)	1.15 (0.86-1.58)	1.33 (0.97-1.85)	1.49 (1.07-2.09)	1.66 (1.18-2.34)	1.97 (1.38-2.79)	<.0001
HDL-C (mmol/L)	1.61 ± 0.40	1.51 ± 0.38	1.43 ± 0.36	1.37 ± 0.35	1.32 ± 0.34	<.0001
LDL-C (mmol/L)	3.55 ± 0.85	3.60 ± 0.86	3.60 ± 0.87	3.58 ± 0.87	3.53 ± 0.87	<.0001
CRP (mmol/L)	1.10 (0.54-2.38)	1.25 (0.62-2.65)	1.36 (0.68-2.81)	1.44 (0.73-2.93)	1.50 (0.76-2.99)	<.0001
	Quintiles of Leucine					
	Q1	Q2	Q3	Q4	Q5	
Age (years)	56.91 ± 8.22	57.10 ± 8.09	57.11 ± 8.04	56.88 ± 8.04	56.77 ± 8.02	<.0001
BMI (kg/m^2^)	25.85 ± 4.61	26.86 ± 4.67	27.54 ± 4.67	28.16 ± 4.65	28.67 ± 4.71	<.0001
SBP (mmHg)	138.20 ± 20.69	139.48 ± 20.09	140.32 ± 19.58	140.75 ± 19.07	140.16 ± 18.54	<.0001
DBP (mmHg)	80.84 ± 10.72	81.95 ± 10.65	82.68 ± 10.63	83.16 ± 10.64	82.41 ± 10.53	<.0001
HbA1c (%)	34.80 (32.40-37.20)	35.00 (32.60-37.50)	35.20 (32.80-37.80)	35.30 (32.90-38.10)	35.60 (33.10-38.60)	<.0001
Total cholesterol (mmol/L)	5.77 ± 1.12	5.76 ± 1.12	5.74 ± 1.12	5.69 ± 1.13	5.61 ± 1.15	<.0001
Triglyceride (mmol/L)	1.14 (0.85-1.60)	1.33 (0.97-1.86)	1.48 (1.07-2.08)	1.65 (1.18-2.32)	1.95 (1.38-2.75)	<.0001
HDL-C (mmol/L)	1.63 ± 0.41	1.52 ± 0.38	1.43 ± 0.36	1.36 ± 0.34	1.31 ± 0.33	<.0001
LDL-C (mmol/L)	3.53 ± 0.85	3.59 ± 0.86	3.61 ± 0.86	3.59 ± 0.87	3.54 ± 0.87	0.4907
CRP (mmol/L)	1.13 (0.54-2.49)	1.27 (0.62-2.70)	1.35 (0.68-2.78)	1.42 (0.72-2.89)	1.47 (0.75-2.89)	<.0001
	Quintiles of Valine					
	Q1	Q2	Q3	Q4	Q5	
Age (years)	56.06 ± 8.35	57.07 ± 8.10	57.21 ± 8.04	57.27 ± 7.95	57.15 ± 7.89	<.0001
BMI (kg/m^2^)	25.46 ± 4.21	26.71 ± 4.46	27.49 ± 4.58	28.30 ± 4.74	29.13 ± 4.96	<.0001
SBP (mmHg)	136.94 ± 20.37	139.42 ± 19.89	140.33 ± 19.53	141.00 ± 19.23	141.24 ± 18.79	<.0001
DBP (mmHg)	80.44 ± 10.63	81.91 ± 10.65	82.61 ± 10.60	83.08 ± 10.64	83.01 ± 10.57	<.0001
HbA1c (%)	34.50 (32.20-37.00)	35.00 (32.60-37.40)	35.20 (32.80-37.80)	35.50 (33.00-38.20)	36.00 (33.30-39.10)	<.0001
Total cholesterol (mmol/L)	5.66 ± 1.08	5.74 ± 1.11	5.74 ± 1.13	5.72 ± 1.15	5.71 ± 1.18	<.0001
Triglyceride (mmol/L)	1.10 (0.84-1.50)	1.31 (0.97-1.79)	1.48 (1.08-2.06)	1.71 (1.22-2.39)	2.08 (1.45-2.98)	<.0001
HDL-C (mmol/L)	1.62 ± 0.39	1.52 ± 0.38	1.44 ± 0.36	1.37 ± 0.35	1.30 ± 0.34	<.0001
LDL-C (mmol/L)	3.47 ± 0.83	3.58 ± 0.86	3.61 ± 0.86	3.62 ± 0.88	3.59 ± 0.88	<.0001
CRP(mmol/L)	1.04 (0.51-2.24)	1.23 (0.61-2.56)	1.35 (0.67-2.78)	1.47 (0.75-2.98)	1.59 (0.82-3.18)	<.0001

BMI, Body Mass Index; SBP, Systolic Blood Pressure; DBP, Diastolic Blood Pressure; HDL-C, High Density Lipoprotein Cholesterol; LDL-C, Low Density Lipoprotein Cholesterin; CRP, C-Reactive Protein.

### 3.2 Incidence of MACE

**Figure 1:**
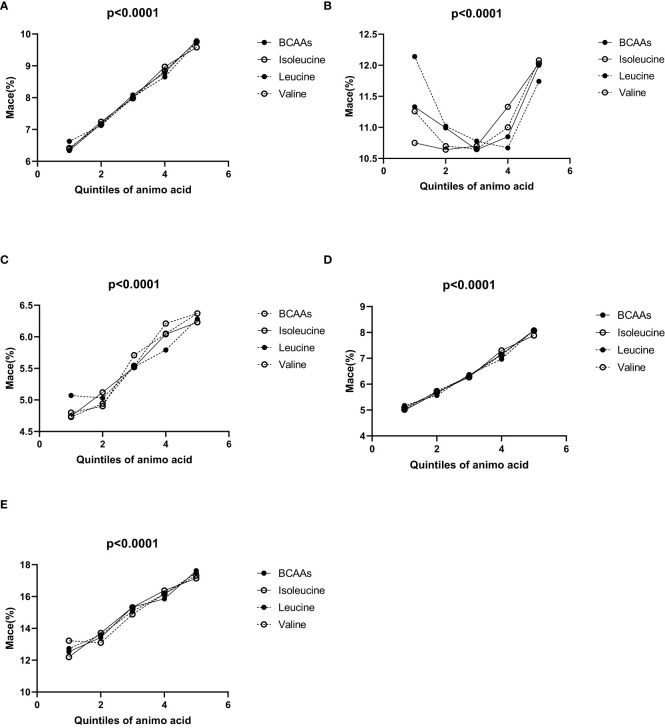
**(A–E)** Incidence of MACE according to the quintiles of BCAAs, isoleucine, leucine and valine. **(A)** Incidence of MACE according to the quintiles of BCAAs, isoleucine, leucine and valine in overall population. **(B)** Incidence of MACE according to the quintiles of BCAAs, isoleucine, leucine and valine in males. **(C)** Incidence of MACE according to the quintiles of BCAAs, isoleucine, leucine and valine in females. **(D)** Incidence of MACE according to the quintiles of BCAAs, isoleucine, leucine and valine in participants under 65. **(E)** Incidence of MACE according to the quintiles of BCAAs, isoleucine, leucine and valine in 65 and older.

In the overall population (**Figure 1A**), the incidence of MACE increased progressively across the quintiles for each BCAA:

- For BCAAs: 6.34% (1st quintile), 7.18% (2nd quintile), 8.08% (3rd quintile), 8.79% (4th quintile), and 9.79% (5th quintile).
- For isoleucine: 6.39% (1st quintile), 7.24% (2nd quintile), 7.99% (3rd quintile), 8.97% (4th quintile), and 9.58% (5th quintile).
- For leucine: 6.63% (1st quintile), 7.13% (2nd quintile), 8.02% (3rd quintile), 8.65% (4th quintile), and 9.74% (5th quintile).
- For valine: 6.42% (1st quintile), 7.16% (2nd quintile), 7.98% (3rd quintile), 8.85% (4th quintile), and 9.76% (5th quintile).

In males (**Figure 1B**), for BCAAs, using the second quintile (10.99%) as a reference, the incidence of MACE was higher in the first quintile (11.33%), and then varied slightly in the third (10.64%), fourth (10.85%), and fifth quintiles (12%). For isoleucine, with the second quintile (10.64%) as a reference, the incidence of MACE was higher in the first quintile (10.75%), and continued to increase in the third (10.70%), fourth (11.33%), and fifth quintiles (12.03%). For leucine, taking the second quintile (11.02%) as a reference, the first quintile showed a higher incidence of MACE (12.14%), while the third (10.78%), fourth (10.67%), and fifth quintiles (11.74%) exhibited variations. For valine, using the second quintile (10.7%) as a reference, the incidence of MACE was higher in the first quintile (11.26%) and increased slightly in the third (10.65%), fourth (11%), and fifth quintiles (12.08%).

In females (**Figure 1C**), for BCAAs, the incidence of MACE increased across the quintiles: 4.8% (1st quintile), 4.9% (2nd quintile), 5.71% (3rd quintile), 6.05% (4th quintile), and 6.37% (5th quintile). For isoleucine, the incidence of MACE rosed gradually across the quintiles: 4.74% (1st quintile), 5.12% (2nd quintile), 5.52% (3rd quintile), 6.04% (4th quintile), and 6.23% (5th quintile). For leucine, the incidence of MACE increased with the quintiles: 5.07% (1st quintile), 5.03% (2nd quintile), 5.52% (3rd quintile), 5.79% (4th quintile), and 6.28% (5th quintile). For valine, the incidence of MACE increased across the quintiles: 4.73% (1st quintile), 4.94% (2nd quintile), 5.54% (3rd quintile), 6.21% (4th quintile), and 6.37% (5th quintile).

In participants under 65 (**Figure 1D**), the incidence of MACE increased progressively across the quintiles for each BCAA:

- For BCAAs: 4.99% (1st quintile), 5.66% (2nd quintile), 6.34% (3rd quintile), 7.15% (4th quintile), and 8.08% (5th quintile).
- For isoleucine: 5.09% (1st quintile), 5.69% (2nd quintile), 6.27% (3rd quintile), 7.30% (4th quintile), and 7.88% (5th quintile).
- For leucine: 5.17% (1st quintile), 5.57% (2nd quintile), 6.37% (3rd quintile), 6.97% (4th quintile), and 8.10% (5th quintile).
- For valine: 5.02% (1st quintile), 5.73% (2nd quintile), 6.30% (3rd quintile), 7.13% (4th quintile), and 8.07% (5th quintile).

In all groups, the p-values were <0.05, indicating statistical significance.

In participants aged 65 and older (**Figure 1E**), the incidence of MACE also increased across the quintiles for each BCAA:

- For BCAAs: 12.54% (1st quintile), 13.42% (2nd quintile), 15.34% (3rd quintile), 15.86% (4th quintile), and 17.62% (5th quintile).
- For isoleucine: 12.20% (1st quintile), 13.71% (2nd quintile), 15.32% (3rd quintile), 16.38% (4th quintile), and 17.14% (5th quintile).
- For leucine: 12.73% (1st quintile), 13.59% (2nd quintile), 15.07% (3rd quintile), 16.16% (4th quintile), and 17.49% (5th quintile).
- For valine: 13.22% (1st quintile), 13.10% (2nd quintile), 14.88% (3rd quintile), 16.16% (4th quintile), and 17.32% (5th quintile).

**Figure 2:**
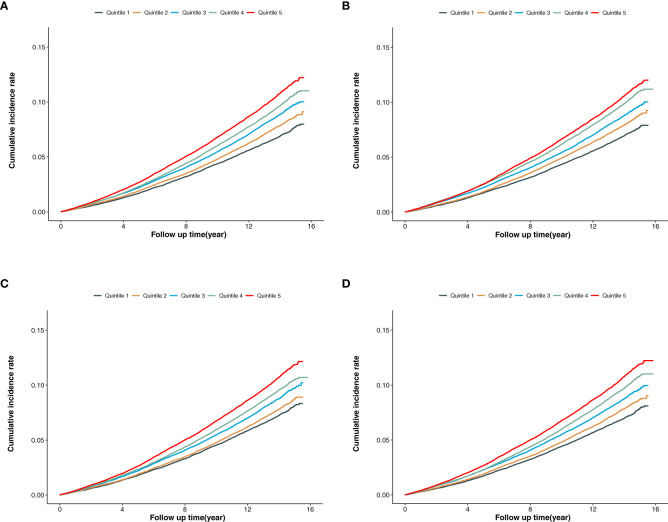
**(A)** Cumulative incidence of MACE according to the quintiles of BCAAs. **(B)** Cumulative incidence of MACE according to the quintiles of isoleucine. **(C)** Cumulative incidence of MACE according to the quintiles of leucine. **(D)** Cumulative incidence of MACE according to the quintiles of valine.

In the overall population (**Figures 2A–D**), the cumulative incidence of MACE was lowest in the first quintile for BCAAs, isoleucine, leucine, and valine, with a gradual increase across the higher quintiles. The log-rank P values for the survival curves in **Figures 2A–D** were all <0.001.

### 3.3 Association between BCAAs and MACE

In the overall population (**Table 2A**), compared to the second quintile, individuals in the fifth quintile of BCAAs had a 7% increased risk of MACE (95% CI 1.02-1.13) after adjusting for age, gender, BMI, HbA1c, LDL, SBP, smoking and drinking status, and physical activity. For isoleucine, the increased risk of MACE was 8% in the fourth quintile (95% CI 1.03-1.14), and 11% in the fifth quintile (95% CI 1.06-1.16). For leucine, participants in the first quintile had a 5% increased risk of MACE (95% CI 1.00-1.11), while those in the fifth quintile had an 8% increased risk (95% CI 1.03-1.13). For valine, the increased risk was 6% in both the first quintile (95% CI 1.01-1.12) and the fifth quintile (95% CI 1.01-1.11).

**Table 2A:** Hazard ratios for associations between MACE and BCAAs in overall population.

	No. of MACE	Rate of 1000	Case/Rate of 1000	Crude HR (95% CI)	Adjusted HR (95% CI)	P value
Quintiles	Overall population					
BCAAs						
Q2	3827	5.512944399	3827/5.51	1.00 (Ref.)	1.00 (Ref.)	
Q1	3393	4.866189389	3393/4.87	0.88 (0.84-0.92)	1.04 (0.99-1.10)	0.1322
Q3	4311	6.231970183	4311/6.23	1.13 (1.08-1.18)	1.01 (0.96-1.06)	0.7911
Q4	4687	6.785146554	4687/6.79	1.23 (1.18-1.29)	1.02 (0.97-1.07)	0.3708
Q5	5222	7.573260286	5222/7.57	1.37 (1.32-1.43)	1.07 (1.02-1.13)	0.0035
Isoleucine						
Q2	3865	5.566568233	3865/5.57	1.00 (Ref.)	1.00 (Ref.)	
Q1	3410	4.884584373	3410/4.88	0.88 (0.84-0.92)	1.03 (0.97-1.08)	0.3294
Q3	4265	6.169475069	4265/6.17	1.11 (1.06-1.16)	1.03 (0.98-1.08)	0.2948
Q4	4788	6.939626693	4788/6.94	1.25 (1.20-1.30)	1.08 (1.03-1.14)	0.001
Q5	5112	7.410744561	5112/7.41	1.33 (1.28-1.39)	1.11 (1.06-1.16)	<.0001
Leucine						
Q2	3806	5.477228949	3806/5.48	1.00 (Ref.)	1.00 (Ref.)	
Q1	3539	5.098394774	3539/5.10	0.93 (0.89-0.97)	1.05 (1.00-1.11)	0.0458
Q3	4280	6.180889455	4280/6.18	1.13 (1.08-1.18)	1.01 (0.96-1.06)	0.7599
Q4	4617	6.680026257	4617/6.68	1.22 (1.17-1.27)	1.00 (0.96-1.06)	0.8505
Q5	5198	7.523889142	5198/7.52	1.37 (1.32-1.43)	1.08 (1.03-1.13)	0.0017
Valine						
Q2	3816	5.495114387	3816/5.50	1.00 (Ref.)	1.00 (Ref.)	
Q1	3437	4.933773993	3437/4.93	0.90 (0.86-0.94)	1.06 (1.01-1.12)	0.0206
Q3	4258	6.155426855	4258/6.16	1.12 (1.07-1.17)	1.00 (0.96-1.06)	0.8525
Q4	4722	6.831947177	4722/6.83	1.24 (1.19-1.30)	1.04 (0.99-1.09)	0.1355
Q5	5207	7.551508749	5207/7.55	1.37 (1.32-1.43)	1.06 (1.01-1.11)	0.0134

Adjusted model:Adjusted for age,gender,BMI,HbA1c,LDL,SBP,smoking and drinking status, physical activity. HR, Hazard ratio.

In males (**Table 2B**), BCAAs didn’t show any significant association with the risk of MACE. For isoleucine, the risk of mace increased by 8% (95% CI 1.02-1.15) in the fourth quintile and by 12% (95% CI 1.05-1.19) in the fifth quintile. For leucine, the risk of mace increased by 9% (95% CI 1.01-1.18) in the first quintile and 6% (95% CI 1.00-1.12) in the fifth quintile. For valine, the risk of mace increased by 8% (95% CI 1.00-1.16) in the first quintile.

**Table 2B:** Hazard ratios for associations between MACE and BCAAs in males.

MACE	No. of MACE	Rate of 1000	Case/Rate of 1000	Crude HR (95% CI)	Adjusted HR (95% CI)	P value
Quintiles	Males					
BCAAs						
Q2	2194	8.667130207	2194/8.67	1.00 (Ref.)	1.00 (Ref.)	
Q1	1432	9.125505822	1432/9.13	1.07 (1.01-1.13)	1.04 (0.96-1.12)	0.3053
Q3	2729	8.359702265	2729/8.36	1.00 (0.95-1.06)	0.95 (0.90-1.02)	0.1476
Q4	3301	8.490216466	3301/8.49	1.02 (0.97-1.08)	0.97 (0.91-1.03)	0.3452
Q5	3888	9.405020018	3888/9.41	1.13 (1.07-1.19)	1.04 (0.98-1.10)	0.2452
Isoleucine						
Q2	2186	8.372945994	2186/8.37	1.00 (Ref.)	1.00 (Ref.)	
Q1	1573	8.513950953	1573/8.51	1.03 (0.97-1.09)	1.06 (0.99-1.14)	0.1192
Q3	2726	8.409756389	2726/8.41	1.05 (0.99-1.10)	1.03 (0.96-1.09)	0.4453
Q4	3351	8.901891594	3351/8.90	1.07 (1.01-1.13)	1.08 (1.02-1.15)	0.0117
Q5	3708	9.452235805	3708/9.45	1.16 (1.10-1.22)	1.12 (1.05-1.19)	0.0003
Leucine						
Q2	2065	8.703454403	2065/8.70	1.00 (Ref.)	1.00 (Ref.)	
Q1	1432	9.834921294	1432/9.83	1.10 (1.04-1.16)	1.09 (1.01-1.18)	0.0282
Q3	2736	8.457309338	2736/8.46	0.95 (0.90-1.01)	0.98 (0.92-1.05)	0.5897
Q4	3339	8.352210074	3339/8.35	1.02 (0.96-1.07)	0.98 (0.92-1.04)	0.4873
Q5	3972	9.182550572	3972/9.18	1.08 (1.03-1.14)	1.06 (1.00-1.12)	0.0651
Valine						
Q2	2192	8.428108826	2192/8.43	1.00 (Ref.)	1.00 (Ref.)	
Q1	1563	9.049615344	1563/9.05	1.04 (0.99-1.10)	1.08 (1.00-1.16)	0.0453
Q3	2717	8.373809497	2717/8.37	1.01 (0.96-1.07)	0.99 (0.93-1.05)	0.7297
Q4	3242	8.595755028	3242/8.60	1.01 (0.96-1.07)	1.00 (0.94-1.06)	0.9707
Q5	3830	9.473554582	3830/9.47	1.14 (1.08-1.20)	1.05 (0.99-1.11)	0.1149

Adjusted model: Adjusted for age,BMI,HbA1c, LDL, SBP, smoking and drinking status, physical activity. HR, Hazard ratio.

In females (**Table 2C**), compared to the second quintile, the third, fourth, and fifth quintiles of BCAAs had an increased risk of MACE by 9% (95% CI 1.00-1.18), 11% (95% CI 1.02-1.21) and 12% (95% CI 1.03-1.22), respectively. For isoleucine, the fourth and fifth quintiles showed increased risks of 10% (95% CI 1.01-1.19) and 9% (95% CI 1.00-1.18), respectively. The fifth quintile of leucine was associated with an 11% (95% CI 1.02-1.20) increased risk. For valine, the third quintile had an increased risk of MACE by 11% (95% CI 1.02-1.20). All findings were adjusted for several potential confounders.

**Table 2C:** Hazard ratios for associations between MACE and BCAAs in females.

MACE	No. of MACE	Rate of 1000	Case/Rate of 1000	Crude HR (95% CI)	Adjusted HR (95% CI)	P value
Quintiles	Females					
BCAAs						
Q2	1633	3.702577646	1633/3.70	1.00 (Ref.)	1.00 (Ref.)	
Q1	1961	3.629214208	1961/3.63	0.99 (0.92-1.06)	1.07 (0.99-1.15)	0.0947
Q3	1582	4.330586064	1582/4.33	1.10 (1.02-1.18)	1.09 (1.00-1.18)	0.0391
Q4	1386	4.589812322	1386/4.59	1.23 (1.15-1.32)	1.11 (1.02-1.21)	0.0121
Q5	1334	4.830969496	1334/4.83	1.29 (1.21-1.39)	1.12 (1.03-1.22)	0.0081
Isoleucine						
Q2	1679	3.875408067	1679/3.88	1.00 (Ref.)	1.00 (Ref.)	
Q1	1837	3.578391936	1837/3.58	0.93 (0.86-1.00)	1.00 (0.93-1.08)	0.9999
Q3	1539	4.191639143	1539/4.19	1.08 (1.00-1.16)	1.03 (0.95-1.11)	0.4644
Q4	1437	4.583530134	1437/4.58	1.19 (1.11-1.28)	1.10 (1.01-1.19)	0.027
Q5	1404	4.718993192	1404/4.72	1.26 (1.17-1.35)	1.09 (1.00-1.18)	0.0479
Leucine						
Q2	1741	3.804509751	1741/3.80	1.00 (Ref.)	1.00 (Ref.)	
Q1	2107	3.841130382	2107/3.84	1.01 (0.94-1.09)	1.04 (0.97-1.12)	0.265
Q3	1544	4.184850853	1544/4.18	1.02 (0.95-1.10)	1.04 (0.96-1.13)	0.3002
Q4	1278	4.385866766	1278/4.39	1.12 (1.05-1.21)	1.05 (0.97-1.14)	0.239
Q5	1226	4.746298523	1226/4.75	1.23 (1.14-1.31)	1.11 (1.02-1.20)	0.0182
Valine						
Q2	1624	3.738894637	1624/3.74	1.00 (Ref.)	1.00 (Ref.)	
Q1	1874	3.576933573	1874/3.58	1.01 (0.94-1.09)	1.06 (0.99-1.15)	0.1059
Q3	1541	4.195671501	1541/4.20	1.16 (1.08-1.25)	1.02 (0.94-1.11)	0.5802
Q4	1480	4.713350545	1480/4.71	1.27 (1.19-1.37)	1.11 (1.02-1.20)	0.011
Q5	1377	4.82738213	1377/4.83	1.38 (1.28-1.48)	1.07 (0.98-1.16)	0.1234

Adjusted model: Adjusted for age,BMI,HbA1c, LDL, SBP, smoking and drinking status, physical activity. HR, Hazard ratio.

In participants younger than 65 (**Table 2D**), compared to the second quintile, the fifth quintile of BCAAs had an increased risk of MACE by 6% (95% CI 1.00-1.13). For isoleucine, the fourth and fifth quintiles showed increased risks of 8% (95% CI 1.02-1.15) and 12% (95% CI 1.06-1.19), respectively. The fifth quintile of leucine was associated with a 6% (95% CI 1.00-1.13) increased risk. Valine did not show an association with the risk of MACE.

**Table 2D:** Hazard ratios for associations between MACE and BCAAs in participants under 60.

MACE	No. of MACE	Rate of 1000	Case/Rate of 1000	Crude HR (95% CI)	Adjusted HR (95% CI)	P value
Quintiles	Participants under 60					
BCAAs						
Q2	2430	4.291746911	2430/4.29	1.00 (Ref.)	1.00 (Ref.)	
Q1	2189	3.777087199	2189/3.78	0.87 (0.82-0.92)	1.03 (0.96-1.10)	0.2709
Q3	2729	4.819793268	2729/4.82	1.12 (1.06-1.19)	0.98 (0.92-1.04)	0.5679
Q4	3095	5.437773564	3095/5.44	1.26 (1.20-1.33)	1.02 (0.96-1.08)	0.4441
Q5	3535	6.162704728	3535/6.16	1.43 (1.36-1.51)	1.06 (1.00-1.13)	0.0398
Isoleucine						
Q2	2446	4.311673922	2446/4.31	1.00 (Ref.)	1.00 (Ref.)	
Q1	2219	3.843043232	2219/3.84	0.89 (0.84-0.94)	1.04 (0.97-1.11)	0.1814
Q3	2707	4.766404339	2707/4.77	1.11 (1.05-1.17)	1.02 (0.96-1.09)	0.5168
Q4	3175	5.559425666	3175/5.56	1.29 (1.22-1.36)	1.08 (1.02-1.15)	0.0079
Q5	3431	6.008799151	3431/6.01	1.39 (1.32-1.47)	1.12 (1.06-1.19)	0.0002
leucine						
Q2	2390	4.216573603	2390/4.22	1.00 (Ref.)	1.00 (Ref.)	
Q1	2226	3.92082602	2226/3.92	0.92 (0.87-0.98)	1.05 (0.98-1.12)	0.1011
Q3	2756	4.842019686	2756/4.84	1.14 (1.08-1.20)	0.99 (0.93-1.05)	0.9536
Q4	3041	5.304934832	3041/5.30	1.26 (1.19-1.32)	0.97 (0.92-1.03)	0.5421
Q5	3565	6.17034723	3565/6.17	1.46 (1.38-1.53)	1.06 (1.00-1.13)	0.0259
Valine						
Q2	2461	4.339959076	2461/4.34	1.00 (Ref.)	1.00 (Ref.)	
Q1	2224	3.802831168	2224/3.80	0.87 (0.82-0.92)	1.00 (0.94-1.07)	0.6901
Q3	2700	4.7854608	2700/4.79	1.10 (1.05-1.17)	0.98 (0.92-1.04)	0.4994
Q4	3076	5.420036644	3076/5.42	1.25 (1.19-1.32)	1.02 (0.96-1.08)	0.5107
Q5	3517	6.15808375	3517/6.16	1.42 (1.35-1.50)	1.05 (0.99-1.11)	0.1018

Adjusted model: Adjusted for gender,BMI,HbA1c, LDL, SBP, smoking and drinking status, physical activity. HR, Hazard ratio.

In participants older than 65 (**Table 2E**), neither BCAAs nor the individual amino acids—isoleucine, leucine, and valine—showed an association with MACE.

**Table 2E:** Hazard ratios for associations between MACE and BCAAs in 60 and older.

MACE	No. of MACE	Rate of 1000	Case/Rate of 1000	Crude HR (95% CI)	Adjusted HR (95% CI)	P value
Quintiles	Participants 60 and older					
BCAAs						
Q2	1397	10.91564915	1397/10.92	1.00 (Ref.)	1.00 (Ref.)	
Q1	1204	10.22826325	1204/10.23	0.93 (0.86-1.00)	1.01 (0.92-1.10)	0.8255
Q3	1582	12.60068816	1582/12.60	1.14 (1.06-1.23)	1.03 (0.95-1.12)	0.3273
Q4	1592	13.09137141	1592/13.09	1.20 (1.12-1.29)	1.01 (0.93-1.10)	0.8839
Q5	1687	14.55319995	1687/14.55	1.32 (1.23-1.42)	1.05 (0.97-1.15)	0.1938
Isoleucine						
Q2	1419	11.1708903	1419/11.17	1.00 (Ref.)	1.00 (Ref.)	
Q1	1191	9.866811717	1191/9.87	0.88 (0.82-0.95)	0.97 (0.89-1.06)	0.5205
Q3	1558	12.62832629	1558/12.63	1.13 (1.05-1.22)	1.03 (0.95-1.12)	0.3751
Q4	1613	13.57190052	1613/13.57	1.22 (1.13-1.31)	1.06 (0.97-1.15)	0.2026
Q5	1681	14.14825049	1681/14.15	1.27 (1.18-1.36)	1.06 (0.98-1.15)	0.1674
Leucine						
Q2	1416	11.05679931	1416/11.06	1.00 (Ref.)	1.00 (Ref.)	
Q1	1313	10.38744952	1313/10.39	0.95 (0.88-1.03)	1.05 (0.96-1.14)	0.4031
Q3	1524	12.36280451	1524/12.36	1.13 (1.05-1.21)	1.02 (0.94-1.11)	0.8034
Q4	1576	13.36441443	1576/13.36	1.22 (1.14-1.32)	1.01 (0.93-1.10)	0.8145
Q5	1633	14.43818797	1633/14.44	1.32 (1.23-1.42)	1.04 (0.96-1.13)	0.4114
Valine						
Q2	1355	10.63754576	1355/10.64	1.00 (Ref.)	1.00 (Ref.)	
Q1	1213	10.8497774	1213/10.85	1.00 (0.92-1.08)	1.09 (0.99-1.18)	0.0522
Q3	1558	12.21593578	1558/12.22	1.14 (1.06-1.23)	1.06 (0.97-1.15)	0.2278
Q4	1646	13.31276232	1646/13.31	1.24 (1.15-1.33)	1.07 (0.98-1.16)	0.0956
Q5	1690	14.27221898	1690/14.27	1.33 (1.24-1.43)	1.07 (0.98-1.16)	0.1603

Adjusted model: Adjusted for gender,BMI,HbA1c, LDL, SBP, smoking and drinking status, physical activity. HR, Hazard ratio.

## 4 Discussion

In this cohort study using the UK Biobank, individuals in the highest quintiles of BCAAs, isoleucine, leucine, and valine had an increased risk of future MACE,except for those aged over 65. This elevated risk was independent of age, gender, BMI, HbA1c, LDL, systolic blood pressure (SBP), smoking and drinking status, and activity level. Additionally, the lowest quintiles of isoleucine and valine also showed elevated MACE risk compared to the second quintiles. In males, higher quintiles of isoleucine, the first and fifth quintiles of leucine, and the first quintile of valine were associated with a higher future risk of MACE. In females, increased MACE risk was observed in the third, fourth, and fifth quintiles of BCAAs; the fourth and fifth quintiles of isoleucine; the fifth quintile of leucine; and the fourth quintile of valine. For individuals under 65, the highest quintiles of BCAAs, isoleucine, and leucine were also associated with a higher risk of MACE. However, in those aged 65 and older, no significant association was found between BCAAs and MACE.

Previous studies have also suggested that high levels of BCAAs are associated with a higher risk of CVD. A nested case-control study conducted by Olle Melander et al. involving 253 pairs of subjects used a baseline AA-score to assess future CVD risk (14). However, this study focused primarily on the DM-AA score and did not address specific amino acid levels. Additionally, the amino acid score only included isoleucine and aromatic amino acids. Another study using data from American and European cohorts indicated that certain amino acids and their gut metabolites might signal the risk of major adverse cardiovascular events (MACE), but it only involved aromatic amino acids (15). A study of 138 heart failure patients found that leucine, valine, and their derivatives predicted mortality risk better than NT-proBNP, though the impact of heart failure itself on amino acid levels remains unclear (13). A study of 700 European Americans (16) suggested a positive correlation between BCAA levels and coronary artery disease (CAD) risk. However, as a cross-sectional study, it could not establish causality. Conversely, a study of 2,346 African Americans found that high leucine levels were associated with a reduced risk of coronary heart disease. This discrepancy may be due to unique socioeconomic factors affecting African Americans, which could potentially influence a range of diseases, including cancer, anxiety, and cardiovascular conditions. Additionally, reduced BCAA levels could be associated with frailty, MACE, and mortality (17).

Our research also conducted subgroup analyses across different gender and age groups. The findings suggests that in males, high levels of isoleucine and leucine, as well as low levels of leucine and valine, are positively correlated with an increased risk of MACE. The subgroup analysis in females reveals that high levels of BCAAs, isoleucine, and leucine are positively correlated with the risk of MACE. Additionally, the fourth quintile of valine, rather than the fifth, was associated with an increased risk of MACE. However, a study involving 27,041 women suggests that the predictive ability of BCAAs for CVD is similar to that of LDL, with isoleucine, leucine, and valine all showing positive association with CVD (18). Additionally, a Finnish study found that isoleucine and leucine are positively associated with cardiovascular events in women, whereas valine did not exhibit a significant correlation (22). Consequently, the association between valine levels and MACE risk in females remains contentious.

The differences in results between genders may be due to the following reasons. Our study suggests that BCAAs are positively correlated with the risk of MACE in the general population. In males, BCAAs levels are negatively correlated with age(the β=-0.04558,p<0.001), meaning that BCAAs levels are lower in elderly males. This may be related to muscle catabolism, which significantly affects circulating BCAAs levels. Muscle atrophy in elderly males and the decrease in BCAAs levels may indicate a more frail state. Therefore, in males, BCAAs are “J”-shaped correlated with the risk of MACE. In contrast, In females included in our study, BCAAs level are positively correlated with age(the β=0.06863,p<0.001). We believed that females inherently have lower muscle mass, and the effect of age-related muscle atrophy on circulating BCAA levels is relatively small. This may explain the differing correlation between BCAAs and MACE risk across genders. Females inherently have lower muscle mass, and the effect of age-related muscle atrophy on circulating BCAA levels is relatively small.

The inconsistent results regarding parity between the US study and ours may be attributed to differences in study period, sample size, and population sociodemographic characteristics, particularly age distribution.

In the subgroup analysis by age, high levels of BCAAs, isoleucine, and leucine were positively correlated with MACE in individuals under 65 years old, but no such correlation was observed in those over 65. Limited research on BCAA levels and aging exists, but one study found that decreased BCAA levels in elderly men over 70 were associated with frailty, MACE, and mortality (23). This discrepancy might be due to a decline in BCAA levels with age (24–26) or frailty (27).

We conducted an analysis in men over 65y and found a J-shaped or J-like correlation between the incidence of MACE and the quintile levels of total BCAAs, isoleucine, leucine, and valine. This could be attributed to muscle catabolism, which has a substantial impact on circulating BCAA levels. The muscle atrophy observed in elderly men, along with the decline in BCAA levels, might signal a more frail state. As a result, in men, BCAAs exhibit a “J”-shaped correlation with the risk of MACE.

Compared to previous studies with smaller sample sizes, our study with a larger sample size more comprehensively demonstrates the correlation between BCAA levels and MACE in men. This does not conflict with previous studies but rather serves as a complement.

Research suggests that in cardiovascular diseases, BCAA metabolism is disrupted due to downregulation of Krüppel-like factor 15 (KLF15), mediated by TAK1 and p38 MAPK signaling (28, 29). This disruption leads to decreased expression of BCAA metabolic enzymes such as BCAT2 (30, 31), BCKDH (10, 12), and PPM1K (32), causing BCAAs and BCKAs to accumulate in the heart. Cardiac injury further disrupts BCAA metabolism in peripheral tissues, increasing circulating BCAA and BCKA levels and their delivery to the heart. Leucine activates mTOR in the heart, inhibiting autophagy through ULK1, promoting insulin resistance via S6K-mediated phosphorylation of insulin receptor substrate 1 (33), and stimulating protein synthesis by phosphorylating 4E-BP1 (34). BCKAs also increase 4E-BP1 phosphorylation and activate the MEK-ERK MAPK pathway, but impair mitochondrial complex I (3, 35), causing oxidative stress. In ischemia-reperfusion injury, Ppm1k deletion leads to BCAA and BCKA accumulation (36), worsening the injury by reducing glucose transport and oxidation (37). In obesity and insulin resistance, BCAA accumulation decreases fatty acid oxidation and increases triglyceride storage. Elevated BCKA levels in obesity and type 2 diabetes impair AKT (38) and PDH in the heart, affecting fuel selection. It’s unclear whether these metabolic changes cause cardiac dysfunction or if cardiac alterations impact BCAA metabolism in other tissues. BCAAs also hinder vascular relaxation through mTOR-dependent ROS generation (39, 40) and promote thrombosis by stimulating tropomodulin 3 propionylation. The valine-derived metabolite 3-HIB (41) increases lipid transport via FATP3 and FATP4. The impact of BCAA-lipid interactions on atherosclerosis is still uncertain.

Our investigation has several strengths. Firstly, it is one of the few large-scale prospective studies to systematically analyze the correlation between branched-chain amino acid levels and the risk of MACE across diverse populations. Secondly, it accounts for lifestyle factors such as glucose and lipid metabolism markers, smoking, alcohol consumption, and physical activity.

However, there are limitations to our study. Firstly, our results are limited to white European participants to avoid genetic heterogeneity, which may restrict the generalizability sof our findings to other ethnic groups. Secondly, the study did not include data on the metabolic products of amino acids and their potential impact on MACE. Future research could address this gap by testing serum samples for these metabolic products and analyzing their correlation with MACE. Lastly, the diagnosis of MACE was based on past medical history, which may have led to the omission of patients with asymptomatic cardiovascular disease.

## 5 Conclusion

Our study consistently observed a positive association between BCAAs, as well as the individual amino acids, isoleucine, leucine, and valine, and the risk of MACE in the overall population. Additionally, a higher risk of MACE was found in females with the lowest quintile of valine and in individuals younger than 65 with the lowest quintile of isoleucine.

## Data Availability

The datasets presented in this study can be found in online repositories. The names of the repository/repositories and accession number(s) can be found below: The UK Biobank data were accessed under application number 96511.
